# Teledialysis in Community Settings: Evaluation of Feasibility, Safety and Effectiveness

**DOI:** 10.1155/ijta/1926027

**Published:** 2026-09-17

**Authors:** Thin Han, Colin Brown, Yishen Wang, Moses Ezeakile, Erin McGovern, Rachel Scott, Zaw Thet, Mahmudul Hassan Al Imam, Gulam Khandaker, Craig F. Munns

**Affiliations:** ^1^ Department of Nephrology, Division of Medicine, Central Queensland Hospital and Health Service (CQHHS), Rockhampton, Queensland, Australia; ^2^ Rural Clinical School, University of Queensland, Rockhampton, Queensland, Australia, uq.edu.au; ^3^ Consultant Nephrologist, Mater Private Hospital Rockhampton, Rockhampton, Queensland, Australia; ^4^ Faculty of Medicine and Dentistry, Griffith University, Gold Coast, Queensland, Australia, griffith.edu.au; ^5^ Central Queensland Public Health Unit, CQHHS, Rockhampton, Queensland, Australia; ^6^ Medical School, The University of Queensland, Brisbane, Queensland, Australia, uq.edu.au; ^7^ Child Health Research Centre, The University of Queensland, Brisbane, Queensland, Australia, uq.edu.au; ^8^ Department of Endocrinology and Diabetes, Queensland Children′s Hospital, Brisbane, Queensland, Australia

**Keywords:** community, haemodialysis, mortality, teledialysis, telemedicine

## Abstract

The number of patients with end‐stage kidney disease requiring haemodialysis (HD) is increasing worldwide, placing substantial pressure on healthcare systems. In Central Queensland, Australia, many patients, including Indigenous Australians, live far from major centres providing HD services. Teledialysis (Tele‐D), involving virtual nephrologist‐led ward rounds, can improve access to specialist care while supporting community‐based dialysis. This study evaluated the feasibility, safety and effectiveness of Tele‐D for satellite HD units in Central Queensland. This retrospective cohort study compared patients receiving in‐centre HD at Rockhampton Hospital (hospital group) with those receiving community HD (Tele‐D group), where dialysis nurses delivered routine care under a nephrologist‐led telehealth model comprising weekly virtual reviews via the Queensland Telehealth Portal and additional telephone or email support as required over a 2‐year period. Both groups received routine monthly pathology review and face‐to‐face outpatient review every 3 months. Clinical and biochemical outcomes, including haemoglobin, urea reduction ratio, albumin, calcium, phosphate and parathyroid hormone, showed no significant differences in longitudinal trajectories between groups. Haemoglobin levels in the Tele‐D group converged towards the recommended target range. Emergency department presentations, hospital length of stay and all‐cause mortality were lower in the Tele‐D group. However, after adjustment for age, sex, diabetes and modified Charlson comorbidity index (mCCI), the hazard ratio for mortality had a confidence interval crossing 1.0, indicating noninferiority rather than superiority. In this regional retrospective cohort, Tele‐D was feasible, with clinical, biochemical and survival outcomes comparable with conventional hospital‐based HD. Because patients in the Tele‐D group were younger, had fewer comorbidities and exclusively used arteriovenous fistulas for vascular access, causal inference cannot be made. These findings support the Tele‐D model of care and warrant further evaluation in larger prospective studies.

## 1. Introduction

Chronic kidney disease (CKD) is one of the leading global public health concerns, with an increasing number of patients requiring renal replacement therapy (RRT) due to an aging population, improved life expectancy for patients with end‐stage renal disease (ESRD) and rise of diabetes mellitus worldwide [1, 2]. Globally, an estimated 7.083 million patients with ESRD require RRT [2]. The majority of RRT is provided through in‐centre/hospital‐based haemodialysis (HD), which is very expensive [1]. The increasing demand for maintenance dialysis therapy is becoming a significant burden on the healthcare systems in many countries [1]. To address this challenge, the promotion of modalities alternative to in‐centre HD, such as community HD or home HD, is desirable. The latter modality has been recommended in the Second Congress of the International Society for HD [1]. However, there are concerns about performing HD outside the hospital setting regarding potential risk of substandard care and outcomes and a perceived lack of medical supervision. These concerns are the most common barriers to setting up home/community HD [3, 4]. However, Teledialysis (Tele‐D), where home/community HD is supervised through videoconferencing, has the potential to overcome these barriers [5]. Despite the potential benefits of Tele‐D in managing patients with ESRD, there is a lack of robust evidence on its use and outcomes in particular at the community setting.

A systematic review identified only three studies reporting on Tele‐D [6]. Rygh et al. [7] reported that home dialysis patients receiving care through Tele‐D are generally satisfied with the remote care, as it facilitates independent living. Whitten and Buis [8] found that all patients from the local dialysis clinics and the providers had positive perceptions of telemedicine. The economic analysis and staff satisfaction were assessed by Rumpsfeld et al. [9]. However, only one study analysed the laboratory parameters and blood pressure as clinical markers [10]. Therefore, more data and outcome assessments are needed to reach definitive conclusions for benefits and risks associated with Tele‐D in the community dialysis units.

The Central Queensland Hospital and Health Services (CQHHS), Australia is a regional area with many dialysis patients living in remote areas far from main dialysis centre (i.e., Rockhampton hospital). A major challenge in this healthcare landscape is to provide adequate dialysis services to the community, especially given the shortage of healthcare workforce. Therefore, we have set up a Tele‐D system, and now, we aim to evaluate the feasibility, safety and effectiveness of the Tele‐D in community dialysis patients in CQHHS.

## 2. Materials and Methods

### 2.1. Study Design and Setting

Rockhampton city has an estimated population of over 84,000 (http://profile.id.com.au/rockhampton/population-estimate), and Rockhampton hospital is the hub hospital in Central Queensland region where the main dialysis unit is situated. The East Street Community Dialysis Centre is 3 km away from the hospital and is an extension of the hospital dialysis service located in the community. This retrospective cohort study compared patients on maintenance HD at the Renal Unit, Rockhampton Hospital (hospital group) with those at the East Street Community Dialysis Centre (Tele‐D group) between June 2021 and May 2023, with follow‐up extending to 36 months. Study inclusion criteria were: adults (≥ 18 years) receiving regular maintenance HD at either site, on HD for at least 6 months, receiving two or more HD sessions per week and having at least 12 months of available HD data. Patients were required to have a minimum dialysis vintage of 6 months to ensure they had reached a stable maintenance phase of HD before study entry. The requirement for at least 12 months of available HD data enabled adequate longitudinal follow‐up and comparable outcome assessment between the two study groups. Exclusion criteria were: holiday HD patient, patients receiving fewer than two HD sessions per week, temporary HD patients (including those with acute kidney injury, satellite unit patients and peritoneal dialysis patients), patients with fewer than 12 months of available HD data, self‐care patients, noncompliant patients, patients who relocated or underwent kidney transplantation during the study period and patients with mental illness or dementia.

### 2.2. Patient Allocation and Eligibility for Tele‐D

Allocation to community Tele‐D or in‐centre hospital dialysis was based on the patient selection criteria as follows: Patients were considered for community Tele‐D if they had an established, well‐functioning vascular access—either an arteriovenous fistula (AVF) or a permanent tunneled CVC (provided the catheter functioned adequately, remained free from infection and appropriate staff training and support were in place); had been clinically stable on dialysis in the preceding 3 months (i.e., stable blood pressure, no ongoing medical complications—especially cardiac complications—dry weight set and stable, no requirements for intensive nursing care, no requirements for medical supervision to undertake dialysis and able to complete the prescribed HD session without ongoing complications); had no unstable mental illness or aggressive/inappropriate behaviours; were independently mobile (with carer support where required) and able to self‐manage between sessions; were geographically located within the CQHHS; and consented to community‐based, virtually supervised care. There were no upper age limits and no exclusion based on diabetes status, number of comorbidities or type of vascular access. Patients requiring hospital‐based dialysis remained in the in‐centre hospital unit. Community‐unit patients were included if they received regular HD at that unit during the study period. Hospital in‐centre patients were included if they received regular HD at the hospital dialysis unit during the same period.

### 2.3. Tele‐D Intervention

Tele‐D was delivered at the East Street community dialysis centre using the Queensland Health Telehealth Portal on the secure Queensland Health network. The supervising nephrologist was based at Rockhampton Hospital, whereas the community dialysis unit was staffed during each dialysis session by renal‐trained registered nurses. Scheduled virtual ward rounds were conducted weekly using an iPad mounted on a mobile trolley, connected to the Telehealth Portal via the centre′s secure Wi‐Fi network.

The virtual ward round was designed to closely replicate a conventional face‐to‐face dialysis ward round closely. Real‐time audio and video communication enabled the nephrologist to review patients individually, assess clinical concerns, inspect vascular access with assistance from on‐site nursing staff, review dialysis machine parameters, ultrafiltration volume and interim biochemistry and discuss management directly with patients and nurses. Headphones or earphones were not routinely used, reflecting standard chairside practice in the open dialysis unit. Consequently, privacy considerations were comparable with routine in‐person dialysis care. All telehealth consultations were conducted using Queensland Health–approved systems in accordance with Queensland Health privacy, information security and telehealth governance requirements.

Between scheduled tele‐rounds, the supervising nephrologist remained available to the on‐site nursing team via dedicated telephone and secure email. Nurses could initiate unscheduled telehealth consultations whenever additional medical review was required. All HD sessions were supervised by experienced dialysis nurses, with routine predialysis assessment including vital signs, weight and symptom review, followed by continuous monitoring throughout treatment.

Any significant clinical deterioration, abnormal observations, dialysis‐related complications or patient concerns prompted immediate nursing assessment and management according to local clinical protocols. Emergency equipment, including resuscitation equipment and emergency medications, was available on‐site, and nursing staff maintained mandatory competency in basic and advanced life support in accordance with Queensland Health standards. No modifications to standard emergency management procedures were required for the Tele‐D model. Nonemergency clinical issues were discussed with the treating nephrologist via telephone or telehealth, whereas patients requiring higher level medical assessment or intervention were transferred to the nearest hospital emergency department (ED) according to established Queensland Health escalation pathways. Patient safety standards therefore remained consistent with conventional hospital‐based HD services.

Clinical and dialysis treatment data were documented in the patient bedside dialysis chart during every treatment session. The chart recorded routine dialysis parameters, observations, prescriptions, clinical issues and treatment changes, providing a shared clinical record between the nephrologist and dialysis nurses to support continuity of care and documentation of clinical decisions. The service operated under CQHHS Renal Service clinical governance with monthly pathology review and quarterly clinical review for all patients.

### 2.4. Data Collection and Outcomes

Laboratory and clinical data (haemoglobin, serum calcium, phosphate, parathyroid hormone [PTH], albumin, urea reduction ratio [URR], ultrafiltration volume, frequency of ED presentations, inpatient length of stay, death and survival) were extracted from the Business Analysis Decision Support (BADS) system, the laboratory information system (test date, test type and test results), and patient files using a pretested Excel spreadsheet. Dialysis parameters were collected from bedside dialysis charts. Comorbidities were extracted from patient files and summarised using the modified Charlson Comorbidity Index (mCCI) [11, 12], categorised as low (0–2), moderate (3–4) or high (≥ 5). Prespecified laboratory targets, consistent with KHA‐CARI [https://www.cariguidelines.org/guidelines/dialysis/biochemical-and-haematological-targets/haemoglobin/] and KDIGO [https://kdigo.org/wp-content/uploads/2026/01/KDIGO-2026-Anemia-in-CKD-Guideline.pdf] guidelines, were as follows: haemoglobin 100–115 g/L; serum phosphate 0.8–1.6 mmol/L; corrected calcium 2.10–2.55 mmol/L; intact PTH 2–9 × upper limit of normal; serum albumin > 35 g/L; single‐pool Kt/V ≥ 1.2 per session; and URR ≥ 65%. Electronic data were held on secure CQHHS servers using password‐protected files; physical documents were stored in locked offices when not in use. Ethics approval (HREC/2023/QCQ/101296) was obtained from the CQHHS Human Research Ethics Committee.

### 2.5. Statistical Analysis

Continuous variables were summarised as mean (SD) and categorical variables as *n* (%); standardised mean differences (SMD) were reported to quantify between‐group imbalance, with |SMD| > 0.10 indicating clinically relevant imbalance. Event outcomes (all‐cause mortality, ED presentations and hospital length of stay) were presented as crude rates per patient‐year with exact Poisson 95% confidence intervals; one‐sided upper bounds were reported where zero events occurred in one arm. Crude incidence rate ratios were reported with exact Poisson confidence intervals.

Survival to 36 months was analysed by the Kaplan–Meier method with a log‐rank test, an unadjusted Cox proportional‐hazards model and a sequential multivariable Cox model with covariates added one at a time (age, sex, diabetes and mCCI score). The proportional‐hazards assumption was assessed using Schoenfeld residuals. Two further sensitivity analyses were performed to address allocation bias: stabilised inverse‐probability‐of‐treatment weighting (IPTW) and 1:1 nearest‐neighbour propensity‐score matching with a 0.2‐SD calliper on the logit of the propensity score. Because the matched cohort exhibited complete separation (all matched‐cohort deaths in the hospital arm), the matched Cox model was fitted using Firth′s penalised likelihood correction. Longitudinal haemoglobin trajectories were modelled with a linear mixed‐effects model (random patient intercept; fixed effects for time, group and their interaction), using all nine quarterly observations per patient. Median follow‐up was estimated by the reverse Kaplan–Meier method. All analyses were conducted in R Version 4.6.0 with two‐sided *α* = 0.05. Given the small sample (*N* = 39, six events), the adjusted survival and propensity‐score analyses were prespecified as exploratory sensitivity analyses, not as primary inferential tests.

## 3. Results

### 3.1. Baseline Characteristics

**Figure 1:**
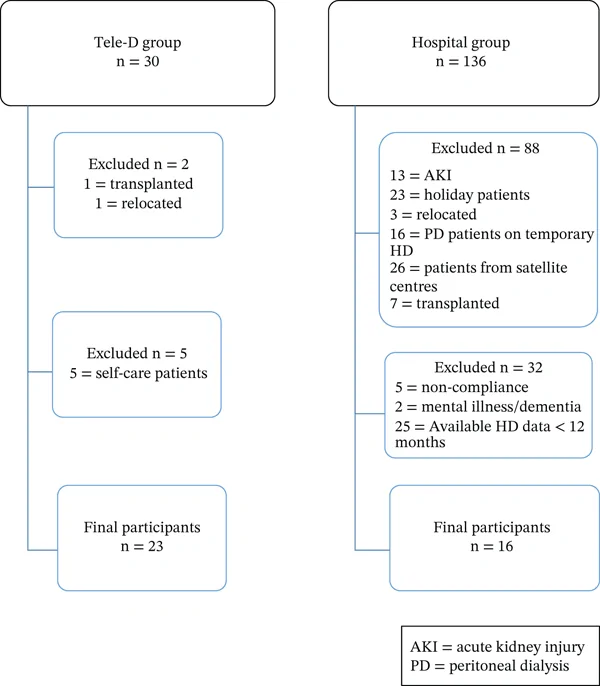
Participant flow chart.

Thirty‐nine patients met inclusion criteria (Tele‐D, *n* = 23; hospital, *n* = 16) (Figure 1); their characteristics are shown in Table 1. The two groups differed substantially at baseline. The Tele‐D cohort was younger (mean 54.7 vs. 67.8 years; SMD 0.89), less likely to be diabetic (26.1% vs. 68.8%; SMD 0.95) and had a lower mCCI score (mean 1.22 vs. 2.62; SMD 1.05). All Tele‐D patients had an AVF, whereas five (31.2%) hospital patients had a permanent central venous catheter (CVC). The primary cause of end‐stage kidney disease was dominated by diabetic nephropathy in the hospital arm (56.2% vs. 26.1% in Tele‐D). Dialysis prescription was broadly similar between groups: most participants dialysed for 3–4 h per session (Tele‐D 88% vs. hospital 87%) and received 2–3 sessions per week; only one Tele‐D patient required more than four sessions per week.

**Table 1:** Demographic and clinical characteristics.

Variable	Level	Overall *N* = 39	Hospital group *n* = 16	Tele‐D group *n* = 23	*p*	SMD
Age, mean (SD)		60.05 (15.99)	67.81 (15.21)	54.65 (14.47)	0.009	0.886
Sex, *n* (%)	F	14 (35.9)	5 (31.2)	9 (39.1)	0.869	0.166
	M	25 (64.1)	11 (68.8)	14 (60.9)		
Indigenous status, *n* (%)	N	30 (76.9)	13 (81.2)	17 (73.9)	0.882	0.177
	Y	9 (23.1)	3 (18.8)	6 (26.1)		
Vascular access						
AVF, *n* (%)		34 (87.2%)	11 (68.8)	23 (100.0)		0.953
Permanent						
CVC, *n* (%)		5 (12.8%)	5 (31.2)	0 (0.0)	0.008	
Dialysis hours, mean (SD)		4.08 (0.44)	4.12 (0.34)	4.04 (0.50)	0.574	0.191
Dialysis sessions/week, mean (SD)		3.10 (0.60)	3.06 (0.25)	3.13 (0.76)	0.732	0.121
Diabetes, *n* (%)	No	22 (56.4)	5 (31.2)	17 (73.9)	0.021	0.945
	Yes	17 (43.6)	11 (68.8)	6 (26.1)		
mCCI score, mean (SD)		1.79 (1.52)	2.62 (1.20)	1.22 (1.48)	0.003	1.045
mCCI category, *n* (%)	Low	26 (66.7)	8 (50.0)	18 (78.3)	0.135	0.617
	Moderate	13 (33.3)	8 (50.0)	5 (21.7)		
Cause of ESKD, *n* (%)	Diabetic nephropathy	15 (38.5)	9 (56.2)	6 (26.1)	0.150	0.663
	Glomerulonephritis	12 (30.8)	3 (18.8)	9 (39.1)		
	Other	12 (30.8)	4 (25.0)	8 (34.8)		
Emergency department visits	39					
0 visits		24 (62%)	1 (6.3%)	23 (100%)	NA	
1–2 visits		6 (15%)	6 (38%)	0 (0%)		
> 2 visits		9 (23%)	9 (56%)	0 (0%)		
Length of stay (h)	39	0 (0, 27)	42 (18, 58)	0 (0, 0)	NA	
Outcome at 36 m	39					
Alive		33 (85%)	11 (69%)	22 (96%)	0.033	
Died		6 (15%)	5 (31%)	1 (4.3%)		

*Note:* |SMD| > 0.10 indicates clinically relevant imbalance. mCCI categorised as low (0–2), moderate (3–4) and high (≥ 5); no patient was in the high category. Glomerulonephritis includes IgA nephropathy, focal segmental glomerulosclerosis, ANCA‐associated glomerulonephritis, anti‐GBM disease, membranous nephropathy and reflux nephropathy.

Abbreviations: AVF, arteriovenous fistula; CVC, central venous catheter; mCCI, modified Charlson comorbidity index; SMD, standardised mean difference.

### 3.2. Follow‐Up and Event Rates

Over a median follow‐up of 36 months (restricted mean 35.0 months in the hospital group, 36.0 months in the Tele‐D group), the total person‐time was 44.3 patient‐years in the hospital group and 67.8 patient‐years in the Tele‐D group (112.1 patient‐years total). The all‐cause mortality rate was 11.3 per 100 patient‐years (95% CI 3.7–26.3) in the hospital group and 1.5 per 100 patient‐years (95% CI 0.04–8.2) in the Tele‐D group, corresponding to a crude incidence rate ratio of 0.13 (95% CI: 0.003–1.17, *p* = 0.039). The all‐cause ED presentation rate was 2.14 per patient‐year (95% CI: 1.73–2.62) in the hospital group; no Tele‐D patient presented to ED during the observation period, giving an exact one‐sided upper 95% bound of 0.044 per patient‐year. Total inpatient bed‐days accumulated at 0.74 days per patient‐year (95% CI: 0.51–1.05) in the hospital group versus zero in the Tele‐D group.

### 3.3. Survival

**Figure 2:**
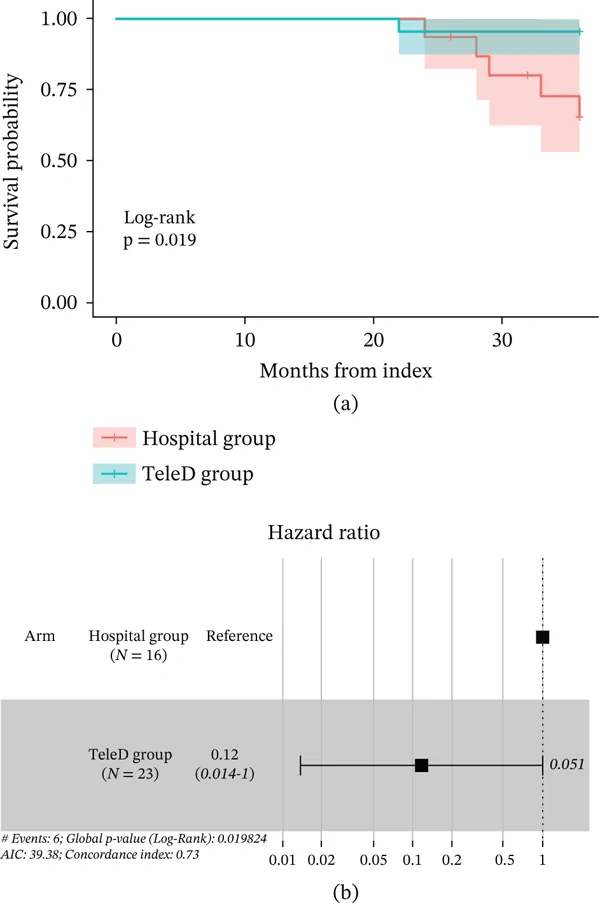
Survival analysis. (a) Kaplan–Meier (KM) curve. (b) Cox proportional hazards model.

Six deaths occurred during the 36‐month observation window (five in the hospital group, one in the Tele‐D group). The Kaplan–Meier survival probability at 36 months was 65.7% (95% CI: 45.3–95.5) in the hospital group and 95.7% (95% CI: 87.7–100.0) in the Tele‐D group; the log‐rank *χ* ^2^ was 5.48 (1 df, *p* = 0.019) (Figure 2a). An unadjusted Cox model gave a hazard ratio (HR) of 0.117 (95% CI: 0.014–1.01), with score and likelihood‐ratio *p* values of 0.02 and a Wald *p* value of 0.051; the Wald *p* value is known to be unreliable with small event counts, so the log‐rank/likelihood‐ratio result is the more appropriate test. Sequential adjustment for confounders in a Cox model produced consistent point estimates but progressively wider confidence intervals: HR: 0.163 (95% CI: 0.016–1.64) with age added, 0.161 (0.016–1.63) with sex added, 0.227 (0.020–2.53) with diabetes added and 0.184 (0.014–2.38) with mCCI score added (Figure 2b; Table S1). The proportional‐hazards assumption was supported (global Schoenfeld *p* = 0.48). Two sensitivity analyses to further address allocation bias—stabilised IPTW and 1:1 nearest‐neighbour propensity‐score matching—yielded HR estimates of 0.501 (95% CI: 0.048–5.27, *p* = 0.57) and 0.114 (95% CI: 0.004–3.56, Firth‐penalised profile‐likelihood *p* = 0.073), respectively; the matched analysis required Firth′s penalised likelihood correction because all three matched‐cohort deaths occurred in the hospital arm (complete separation). Covariate balance before and after IPTW is shown in Figure S1. Across all analyses, the point estimate for the Tele‐D versus hospital HR remained in the 0.11–0.50 range, but the confidence interval crossed 1 in every adjusted model.

### 3.4. Biochemical and Dialysis‐Adequacy Outcomes

Biochemical parameters over the 24‐month observation window are summarised in Table 2. The mean haemoglobin in the Tele‐D group was 123.2 g/L (SD 16.5) at baseline (June 2021) and 115.8 g/L (SD 16.8) at endline (June 2023), representing a within‐group change of −9.6%. The corresponding values in the hospital group were 111.2 g/L (SD 21.9) at baseline and 105.2 g/L (SD 20.7) at endline (change −5.4%). The Tele‐D group had a higher mean haemoglobin at the baseline, exceeding the upper limit of the prespecified target range (100–115 g/L), but values progressively converged towards the target over follow‐up, whereas the hospital group remained within the target throughout. To make use of all nine quarterly haemoglobin measurements per patient rather than only the baseline and endline values, we fitted a linear mixed‐effects model on the 345 patient‐quarter observations. The model showed parallel downward trajectories of −0.17 g/L per month (95% CI: −0.42 to 0.08) in the hospital group and −0.28 g/L per month (95% CI: −0.49 to −0.06) in the Tele‐D group, with no statistically significant difference in slope between groups (group × time interaction *p* = 0.52). The observed convergence of haemoglobin in the Tele‐D group from above the guideline target range towards the target range reflects a reduction from the initially higher baseline values. Given the absence of a significant group × time interaction, this finding should not be interpreted as evidence of poor anaemia outcomes or a clinically meaningful difference between the two models of care. Other biochemical parameters (calcium, phosphate, PTH, albumin, URR and ultrafiltration volume) showed no statistically significant between‐group differences in trajectory (all interaction *p* > 0.05; Table S2).

**Table 2:** Laboratory parameter.

Variable	Baseline (June 21) mean (SD)	Endline (June 23) mean (SD)	Within‐group % change	Between‐group change, mean difference [95% CI] at endline^a^	*p* (between group change)
Haemoglobin					
Tele‐D	123.2 (16.5)	115.8 (16.8)	−9.6	4.8 [−5.3, 15.0]	< 0.001
Hospital	111.2 (21.9)	105.2 (20.7)	−5.4		
Serum calcium					
Tele‐D	2.4 (0.2)	2.4 (0.2)	0.0	−0.04 [−0.2, 0.1]	0.628
Hospital	2.2 (0.1)	2.4 (0.2)	9.1		
Serum phosphate					
Tele‐D	1.9 (0.4)	1.9 (0.5)	0	0.1 [−0.2, 0.5]	0.489
Hospital	1.7 (0.6)	1.6 (0.6)	−5.9		
Serum albumin					
Tele‐D	36.9 (4.3)	33.8 (3.3)	−15.8	1.0 [−1.0, 2.9]	0.315
Hospital	35.0 (4.1)	32.9 (2.6)	−6.0		
Parathyroid hormone					
Tele‐D	39.7 (53.5)	71.8 (71.0)	80.9	−8.6 [−58.9, 41.7]	0.729
Hospital	39.3 (19.8)	80.9 (88.2)	105.9		
Urea reduction ratio					
Tele‐D	70.9 (7.8)	74.0 (8.3)	4.4	0.4 [−5.0, 5.8]	0.876
Hospital	68.7 (5.8)	72.3 (7.1)	5.2		
Ultrafiltration					
Tele‐D	3.1 (1.0)	3.0 (1.1)	−3.2	0.7 [−0.02, 1.5]	0.055
Hospital	3.1 (0.8)	2.4 (1.1)	−22.6		

^a^Analysis of covariance keeping baseline scores as covariates and arm as a fixed factor.

## 4. Discussion

In this retrospective cohort study, we evaluated the feasibility, safety and effectiveness of Tele‐D as an alternative to in‐centre HD for patients managed at a satellite community dialysis unit in Central Queensland, Australia. Over 36 months of follow‐up, patients allocated to Tele‐D achieved clinical and biochemical outcomes comparable with those managed in the hospital setting, with no ED presentations across 67.8 patient‐years of follow‐up and a lower crude mortality rate (1.5 vs. 11.3 per 100 patient‐years). However, after adjustment for age, sex, diabetes and the mCCI in a sequential Cox model, the HR for mortality (0.184; 95% CI: 0.014–2.38) had a confidence interval crossing 1 and propensity‐score sensitivity analyses produced concordant estimates (stabilised IPTW HR 0.501; 1:1 propensity‐score matched HR 0.114 with Firth′s correction). These findings support the feasibility and noninferiority of Tele‐D, delivered by experienced on‐site renal nurses under the supervision of a remotely located nephrologist, while indicating that the observed survival advantage cannot be attributed causally to the model of care because of baseline differences between the groups.

Clinical and biochemical outcomes were comparable between the Tele‐D and hospital groups throughout follow‐up. Haemoglobin levels in the Tele‐D group, although initially slightly above the recommended target range, decreased significantly over time and remained within target levels, whereas the remaining laboratory parameters showed no significant differences between the two groups. These findings suggest that routine clinical management, including anaemia and biochemical control, can be maintained effectively through a Tele‐D model.

The Tele‐D model also demonstrated an excellent safety profile. No patients managed through Tele‐D required ED presentation or hospital admission during the study period. Although the relatively short follow‐up and modest sample size may have limited the detection of infrequent adverse events, these findings support the safety of managing clinically stable HD patients within a community‐based dialysis unit supervised remotely by an experienced nephrologist.

At 36 months, crude mortality was significantly lower in the Tele‐D group than in the hospital group (4.3% vs. 31.0%). However, important baseline differences existed between the groups. Patients allocated to Tele‐D were substantially younger (mean 54.6 vs. 67.8 years), had a lower prevalence of diabetes (26% vs. 69%), lower comorbidity burden (mean mCCI 1.22 vs. 2.62) and all dialysed through a mature AVF, whereas almost one‐third of hospital patients used a permanent CVC. Although all patients in the Tele‐D community cohort had functioning AVFs, this was not a predefined study inclusion criterion or a requirement for community dialysis. Indeed, local service policy permitted patients with tunneled permanent CVCs to receive community dialysis under defined clinical conditions; however, no such patients were present in the community dialysis service during the study period. Therefore, the observed difference in vascular access reflects the characteristics of the service population rather than intentional selection based on vascular access type. In contrast, the hospital cohort included the full spectrum of maintenance HD patients, including those with permanent CVC due to delayed fistula maturation, failed or unsuitable vascular access, previous access complications or other clinical considerations. The use of permanent CVC, however, has been reported to be associated with higher mortality in HD patients [13]. Adjustment for these prognostic factors substantially attenuated the apparent survival advantage, with confidence intervals crossing unity in both multivariable and propensity‐score analyses. Consequently, the observed mortality difference should not be interpreted as evidence that Tele‐D itself improves survival. Rather, the findings indicate that carefully selected, clinically stable patients can safely receive dialysis within a Tele‐D model without compromising clinical outcomes.

Previous studies have suggested that telemedicine‐supported dialysis may improve outcomes by facilitating longer dialysis sessions, which have been associated with lower mortality in HD populations and may partly explain international differences in survival [14–16]. However, dialysis treatment times were similar between the two groups in our study, making this mechanism unlikely to account for the observed mortality difference. Instead, the most plausible explanation lies in the marked baseline differences between the study cohorts, particularly age, diabetes, comorbidity burden, vascular access and the unavoidable residual confounding associated with pragmatic clinical allocation. Vascular access could not be included in the adjusted models because all Tele‐D patients dialysed via AV fistulas. Therefore, the independent contribution of vascular access to the observed outcomes could not be evaluated.

Our study is one of the few evaluating telemedicine‐supported HD delivered within a community satellite dialysis unit. Strengths include complete capture of ED presentations and hospital admissions within the CQHHS through the BADS system, systematic quarterly biochemical monitoring for all patients, a prespecified statistical adjustment strategy and transparent reporting of the patient selection process. These features provide robust evidence supporting the feasibility of Tele‐D in routine regional clinical practice.

Several limitations should be acknowledged. First, allocation to Tele‐D reflected routine clinical practice rather than randomisation, with patients transferred to the community unit only after demonstrating dialysis stability. Consequently, the Tele‐D cohort was younger, had a lower comorbidity burden, had fewer patients with diabetes and all dialysed via AVF. Although vascular access was not a criterion for community dialysis eligibility, these baseline differences are inherent to observational comparisons and likely contributed to the favourable outcomes observed in the Tele‐D cohort. At the same time, they reflect the clinical characteristics considered necessary for safe community dialysis. Second, despite adjustment using multivariable Cox regression and propensity‐score analyses, the small sample size (*N* = 39) and limited number of deaths (*n* = 6) resulted in statistically fragile estimates, with reduced effective sample size after weighting and complete separation requiring Firth′s penalised likelihood correction in the matched analysis. Third, ED presentations and hospital admissions were identified through the BADS database and therefore did not capture presentations to interstate or private hospitals. Fourth, detailed information on ESA, intravenous iron and CKD‐MBD therapies was not available; therefore, differences in biochemical parameters should be interpreted as observed clinical outcomes rather than treatment effects. Finally, this was a single‐centre study from a regional Australian health service, which may limit generalisability to other healthcare settings.

Overall, our findings demonstrate that Tele‐D is a feasible, safe and clinically effective model of care for carefully selected patients receiving maintenance HD in a community satellite unit. Although the lower observed mortality appears largely explained by favourable baseline patient characteristics rather than the Tele‐D intervention itself, the comparable clinical outcomes, excellent safety profile and absence of increased healthcare utilisation support Tele‐D as a practical alternative to conventional hospital‐based HD. Larger prospective, preferably registry‐linked studies are warranted to confirm these findings and determine whether telemedicine‐based HD can influence long‐term clinical outcomes beyond providing an effective alternative model of care.

## 5. Conclusion

Tele‐D is a promising alternative model of HD care that can be integrated into routine clinical practice. With increasing demand for HD services, it may provide a practical and scalable approach for health services facing workforce and resource constraints. In this clinically selected cohort, Tele‐D appeared feasible and safe, with no evidence of worse clinical, biochemical or survival outcomes than conventional hospital‐based HD. Although these findings are encouraging, they should be interpreted in the context of the study′s observational design and clinically selected population. Further evaluation in larger, adequately powered prospective studies is required to confirm the safety, effectiveness and generalisability of this model of care. Based on our clinical experience and the available literature, we propose pragmatic patient selection criteria to support implementation of community Tele‐D (Table 3). These recommendations are intended as service‐level guidance and require prospective validation before widespread adoption.

**Table 3:** Proposed pragmatic patient selection criteria for community Tele‐D.

Domain	Suggested selection criteria
Vascular access	An established, well‐functioning vascular access, preferably a mature AVF, or in selected patients, a stable AVG, with no recurrent access‐related complications during the preceding 3 months. (Patients dialysing via permanent CVCs were not represented in our Tele‐D cohort, although not the exclusion criterion. Consequently, the suitability of Tele‐D for patients with permanent CVCs remains unclear and required further evaluation).
Dialysis stability	Clinically stable on haemodialysis for at least 3 months, including stable blood pressure, stable dry weight, no ongoing medical complications (particularly cardiac complications), no requirement for intensive nursing care or direct medical supervision during dialysis and the ability to complete prescribed dialysis sessions without ongoing complications.
Comorbidity and frailty	Comorbidity burden and frailty within a range that can be safely managed by the on‐site nursing team between scheduled tele‐rounds. (In our cohort, a mCCI in the low category (0–2) was characteristic of most Tele‐D patients; however, this should not be interpreted as a validated threshold).
Mobility and transport	Adequate mobility and reliable transport to the satellite dialysis unit, independently or with carer support.
Cognitive and functional capacity	Able to participate in tele‐consultations, understand clinical advice and undertake appropriate self‐management.
Patient preference	Willingness to receive community‐based dialysis with virtual nephrologist review, supported by informed consent.
Geographic considerations	Reasonable proximity to the satellite dialysis unit, with timely access to ambulance services and an acute hospital should escalation of care be required.

*Note:* These criteria are proposed as pragmatic service‐level guidance based on our clinical experience, study findings and published literature. They are intended to assist patient selection for community Tele‐D and should be regarded as hypothesis‐generating until validated in prospective multicentre studies.

Abbreviations: AVF, arteriovenous fistula; AVG, arteriovenous graft; CVC, central venous catheter; mCCI, modified Charlson comorbidity index.

## Funding

This study was supported by RRGP (Research Ready Grant Program), 101296.

## Conflicts of Interest

The authors declare no conflicts of interest.

## Supporting information


**Supporting Information** Additional supporting information can be found online in the Supporting Information section. Table S1 presents the sequentially adjusted Cox proportional‐hazards models for the primary outcome. Table S2 presents the longitudinal biochemical trajectories fitted using linear mixed‐effects models. Figure S1 shows covariate balance before and after stabilised inverse‐probability‐of‐treatment weighting.

## Data Availability

Data that support the findings of this study are available from the corresponding author upon request. The data are not publicly available due to privacy or ethical restrictions.
