# Supplementary material for: Teledialysis in Community Settings: Evaluation of Feasibility, Safety and Effectiveness
Source: Int J Telemed Appl. 2026 Sep 17;2026:1926027. doi: 10.1155/ijta/1926027 (PMC13586554; doi:10.1155/ijta/1926027)
Supplement: Supplementary file 1 [file IJTA-2026-1926027-s001.docx]

Supplementary Table 1. **Sequentially adjusted Cox proportional hazards models for the primary outcome**

| **model** | **term** | **HR** | **LCL** | **UCL** | **p** | **covariates** | **n_used** | **events** | **EPV** |
| --- | --- | --- | --- | --- | --- | --- | --- | --- | --- |
| m0 | groupTeleD group | 0.117 | 0.014 | 1.009 | 0.051 |  | 39 | 6 | 6 |
| m1 | groupTeleD group | 0.163 | 0.016 | 1.637 | 0.1232 | age | 39 | 6 | 3 |
| m2 | groupTeleD group | 0.161 | 0.016 | 1.628 | 0.1218 | age + sex | 39 | 6 | 2 |
| m3 | groupTeleD group | 0.227 | 0.02 | 2.528 | 0.2279 | age + sex + diabetes | 39 | 6 | 1.5 |
| m4 | groupTeleD group | 0.184 | 0.014 | 2.382 | 0.1952 | age + sex + diabetes + mCCI score | 39 | 6 | 1.2 |

**Abbreviations:** HR, hazard ratio; LCL, lower 95% confidence limit; UCL, upper 95% confidence limit; mCCI, modified Charlson Comorbidity Index; EPV, events per variable. EPV was calculated as the number of outcome events divided by the number of predictor variables included in each model.

Supplementary Table 2. **Longitudinal biochemistry trajectories fitted by linear mixed-effects model**

Supplementary Figure 1. **Covariate balance before and after stabilized inverse probability of treatment weighting (IPTW).**

*Absolute mean differences for baseline covariates are shown before(unadjusted) and after (adjusted) weighting. The vertical dashed line indicates the conventional threshold for acceptable balance (absolute mean difference <0.1)*
